# Smoking, obstructive sleep apnea syndrome and their combined effects on metabolic parameters: Evidence from a large cross-sectional study

**DOI:** 10.1038/s41598-017-08930-x

**Published:** 2017-08-18

**Authors:** Huaming Zhu, Huajun Xu, Rui Chen, Suru Liu, Yunyan Xia, Yiqun Fu, Xinyi Li, Yingjun Qian, Jianyin Zou, Hongliang Yi, Jian Guan

**Affiliations:** 1 Department of Respiratory Medicine, the Second Affiliated Hospital of Soochow University, 1055 Sanxiang Road, 215004 Suzhou, China; 2 Sleep Center, The Second Affiliated Hospital of Soochow University, 1055 Sanxiang Road, 215004 Suzhou, China; 3 Department of Otolaryngology Head and Neck Surgery & Center of Sleep Medicine, Shanghai Jiao Tong University Affiliated Sixth People’s Hospital, 600 Yishan Road, 200233 Shanghai, China; 4 Otolaryngological Institute of Shanghai Jiao Tong University, 600 Yishan Road, 200233 Shanghai, China

## Abstract

Metabolic disorders have been separately associated with obstructive sleep apnea syndrome (OSAS) and smoking. However, no study has examined their interactions with metabolic parameters, including insulin resistance and dyslipidemia. To investigate whether the combination of OSAS and smoking results in an additive detriment in metabolic disorder parameters, we enrolled consecutive adult men during 2014–2015. Fasted blood samples were taken to determine glucose, insulin, and lipid levels. A questionnaire including an item on smoking pack-year exposure was administered, and the Epworth Sleepiness Scale and overnight polysomnography were performed. Smokers showed higher levels of glucose, insulin, total cholesterol (TC), triglycerides (TG), and low density lipoprotein-cholesterol (LDL-C), but lower high-density lipoprotein cholesterol (HDL-C) levels, than did non-smokers. In addition, the risks for insulin resistance increased with OSAS severity without fully adjustment. An OSAS × smoking interaction was found in insulin resistance after adjusting for potential confounding factors (*p* = 0.025). Although the difference was not significant, cessation of cigarette smoking seems to have a little benefit for smoking patients with OSAS. A synergistic effect was observed between smoking and OSAS on metabolic disorder parameters. Cessation of cigarette smoking may experience minor benefit for insulin resistance and lipid metabolism in patients with OSAS.

## Introduction

Obstructive sleep apnea syndrome (OSAS) and smoking are important global health problems. OSAS affects about 10–50% of middle-aged men and prevalence increases with age and obesity^1, 2^. Smoking also affects many people, and the number of smokers worldwide is increasing annually^3^. Both OSAS and smoking are considered traditional risk factors for cardiovascular morbidities^4^.

The association between dyslipidemia, which is an important risk factor for cardiovascular diseases (CVDs), and OSAS has been studied^5^. Several clinical studies have revealed that OSAS is independently associated with particular lipids and that continuous positive airway pressure (CPAP) therapy can reverse the dyslipidemic status of patients with OSAS^6, 7^. Similarly, OSAS is independently associated with impaired glucose metabolism, including fasting hyperglycemia and insulin resistance, and CPAP has a favorable effect on insulin resistance^8, 9^. Several pathophysiological mechanisms, such as elevated sympathetic activity, systemic inflammation, oxidative stress, and sleep fragmentation, may contribute to the occurrence of metabolic disorders (i.e., abnormal lipid and glucose metabolism) in patients with OSAS^10, 11^. The prevalence of smoking is high among patients with OSAS when compared with patients who do not have OSAS^12^. In addition, male OSAS patients smoke more than female OSAS patients^13^. Similar to OSAS, smoking is a well-recognized and important risk factor for metabolic disorders^14, 15^. Various compounds in cigarette smoke, such as volatile organic compounds, heavy metals, and nicotine, increase oxidative stress and systemic inflammation, which play roles in the occurrence of metabolic disorders^16^.

As OSAS and smoking often co-occur, they may share common oxidative and inflammatory pathways that contribute to lipid and glucose metabolic dysfunction^4^. Several previous cross-sectional studies have shown that the interaction between smoking and OSAS leads to increased endothelial dysfunction^17^, cognitive impairment^18^, and cardiovascular risk^19^. However, no study has explored the interaction between OSAS and smoking on glucose and lipid abnormalities. In this study, we enrolled a large cross-sectional cohort and investigated whether tobacco smoking synergized with OSAS to aggravate metabolic profile abnormalities, and whether stopping smoking improves metabolic disorder in patients with untreated OSAS.

## Materials and Methods

### Study population

Chinese adult men were enrolled consecutively from the Sleep Centers in Shanghai Jiao Tong University Affiliated Sixth Hospital and the Second Affiliated Hospital of Soochow University between June 2014 and June 2015. All participants underwent overnight polysomnography (PSG). Written informed consent was obtained from each participant. This study was approved by the Internal Review Board of the Institutional Ethics Committee of the Shanghai Jiao Tong University Affiliated Sixth Hospital and the Second Affiliated Hospital of Soochow University, which was conducted in accordance with the Declaration of Helsinki.

The inclusion criteria were: (1) male gender; (2) suffering from habitual snoring and/or daytime sleepiness; and 3) age of 18–65 years. Exclusion criteria were: (1) other sleep disorders, such as central sleep apnea, upper airway resistance syndrome, restless leg syndrome, rapid eye movement sleep behavior disorders, and narcolepsy; (2) history of medication use for hypertension, diabetes mellitus, or hyperlipidemia; (3) previous treatment for OSAS, such as upper airway surgery or CPAP intervention; (4) systemic diseases (i.e., hepatic, pulmonary, renal, or cardiac failure); and (5) inability to provide written informed consent.

### Anthropometric and blood pressure measurements

Standardized anthropometric parameters, including waist circumference (WC), hip circumference (HC) and neck circumference (NC), were measured using standard methods as described previously^6^. These anthropometric data were recorded as the mean of two measurements. Body mass index (BMI) was calculated as weight divided by height squared (kg/m^2^). Waist-hip ratio (WHR) was calculated as waist circumference divided by hip circumference.

Daytime systolic and diastolic blood pressure was measured at 8 a.m. using a mercury sphygmomanometer, with participants in a seated position after a 5-min rest; the mean of three measurements was recorded.

### Smoking history

When designing the study, one of the authors designed a smoking questionnaire, which comprehensively referenced a smoking survey conducted by the Division of Tobacco Control and Prevention, China Center for Disease Control and Prevention^20^. The smoking questionnaire included smoking status (non-smoker, current smoker, and ex-smoker), mean number of cigarettes smoked per day, smoking duration (years), and number of years since quitting for ex-smokers. Non-smokers were defined as subjects who had never smoked cigarettes. Smokers were defined as self-reported smokers who smoked cigarettes for at least 12 months. Ex-smokers were former cigarette smokers who gave up after smoking for at least 12 months and remained abstinent for at least 12 months. For details of the smoking questionnaire, please see the supplemental material.

### Biochemical measurements

Fasting venous blood was collected at 8 a.m. the morning after PSG. Plasma glucose, insulin, and lipid levels [high-density lipoprotein cholesterol (HDL-C), low density lipoprotein-cholesterol (LDL-C), total cholesterol (TC) and triglycerides (TG)] were measured as described previously^21^. The homeostasis model assessment-insulin resistance (HOMA-IR) was calculated using the following formula: HOMA-IR = fasting serum insulin (μU/mL) × fasting plasma glucose (mmol/L)/22.5^22^. We defined insulin resistance values exceeding the median HOMA-IR value as abnormal, and values below the median were deemed normal. Dyslipidemia of TC, TG, HDL-C, and LDL-C was defined as ≥5.17, ≥1.7, <1.03, and ≥3.33 mmol/L, respectively, according to the diagnostic criteria of the US National Cholesterol Education Program Adult Treatment Panel III^23^.

### Epworth Sleepiness Scale (ESS) questionnaire and PSG monitoring

The self-administered ESS questionnaire is used widely in China as a measure of sleep propensity; it is reliable and has been validated^24, 25^. The ESS consists of eight questions (each scored on a scale ranging from 0 to 3) with total scores ranging from 0–24.

Objective sleep status was evaluated by in-laboratory PSG (Alice 5; Respironics, Pittsburgh, PA, USA). We used this standard PSG to assess the nocturnal sleep of each participant. This PSG utilizes electroencephalogram (EEG) channels (C3-M2 and C4-M1), electrocardiography (ECG), left/right electrooculography (EOG), chin electromyography (EMG), nasal and oral airflow (using both a nasal-oral thermocouple and nasal pressure cannula), snoring sounds, thoracic/abdominal movements, figure pulse oxygen saturation, leg movements, and body position to measure various sleep parameters. Apnea, hypopnea, oxygen desaturation, micro-arousals and the Apnea/Hypopnea Index (AHI), Oxygen Desaturation Index (ODI), and Micro-Arousal Index (MAI) were scored automatically using computer software and subsequently checked manually by a skilled technician, following the American Academic Sleep Medicine (AASM) 2007 criteria^26^. Apnea was defined as cessation in airflow of at least 90% of baseline lasting for at least 10 s; hypopnea was defined as a ≥50% decrease in airflow for 10 seconds or longer, associated with a ≥3% oxygen desaturation or an arousal; arousals were identified as abrupt shifts in electroencephalographic frequency lasting at least 3 s^26^. The AHI was defined as the number of apnea and hypopnea events per hour of sleep; the ODI was defined as the total number of episodes of oxyhemoglobin desaturation ≥4% per hour of sleep; and the MAI was defined as the mean number of arousals per hour of sleep. The severity of OSAS was defined based on the AHI and was categorized as normal (<5), mild (5–14.9), moderate (15–29.9), or severe (≥30)^26^.

### Statistical analysis

The raw data were first examined by the Kolmogorov–Smirnov test and presented as means ± standard deviation, medians (interquartile range), and numbers (percentage) if they were normally distributed, skewed, or categorical. Differences in normally distributed demographic and clinical variables among the three groups were analyzed by analysis of variance (ANOVA) or the Kruskal–Wallis test if they were not normally distributed. Continuous variables were compared using the *t*-test or Mann–Whitney *U* test, and numerical data were compared using Pearson χ^2^ or Fisher’s exact test. *P*-values for linear trends across different groups were calculated using the polynomial linear trend test for continuous variables. Relationship between sleep variables and lipid profile were analyzed by partial correlation analysis and stepwise multiple regression analyses. Metabolic parameters among different OSAS severity and smoking groups were analyzed by two-way ANOVA with the Bonferroni post-hoc test. We also employed analysis of covariance after adjusting for age, BMI, WHR, and ESS. All statistical analyses were performed using SPSS software (ver. 20.0; SPSS Inc., Chicago, IL, USA). A p-value < 0.05 was considered significant.

## Results

### Baseline characteristics of the participants

This study enrolled 719 Chinese men who were habitual snorers. First, we stratified the subjects into two groups: ever-smokers and non-smokers. In both groups, the patients with OSAS were older, more obese, and had worse glucose metabolism, and higher TG and lower HDL-C levels compared with non-OSAS patients. The baseline demographic and clinical characteristics of the subjects are presented in Table 1. Further, we stratified the participants according to OSAS severity. Of the 719 subjects, 138 did not have OSAS (87 non-smokers and 51 ever-smokers), 186 had mild OSAS (103 non-smokers and 83 ever-smokers), 196 had moderate OSAS (100 non-smokers and 96 ever-smokers), and 199 had severe OSAS (91 non-smokers and 108 ever-smokers). In total, 381 subjects (53.0%) were classified as non-smokers, 59 (8.2%) were ex-smokers, and 279 (38.8%) were active smokers (Table 2). *P*-values for linear trends across different OSAS severity groups were calculated using the polynomial linear trend test for continuous variables. We found that increasing AHI was associated with increasing glucose, insulin, HOMA-IR, and TG levels, and with decreasing HDL-C levels (linear trend, all *p* < 0.05).

**Table 1:** Demographic and clinical characteristics of the enrolled subjects stratified by smoking status.

	Ever-smokers			Non-smokers		
	non-OSAS (n = 51)	OSAS (n = 287)	p value	non-OSAS (n = 87)	OSAS (n = 294)	p value
Age, years	38.65 ± 11.14	42.16 ± 10.00	0.02	35.60 ± 10.98	41.32 ± 12.21	<0.01
BMI, Kg/m^2^	24.61 ± 3.01	26.88 ± 3.18	<0.01	23.83 ± 2.41	26.08 ± 3.08	<0.01
NC, cm	38.29 ± 2.40	39.97 ± 2.47	<0.01	37.82 ± 2.22	39.27 ± 2.56	<0.01
WC,cm	89.21 ± 7.89	96.16 ± 8.39	<0.01	86.86 ± 7.65	93.76 ± 7.91	<0.01
HC,cm	97.29 ± 5.56	100.7 ± 6.23	<0.01	96.45 ± 5.75	99.70 ± 6.28	<0.01
WHR	0.92 ± 0.04	0.95 ± 0.05	<0.01	0.90 ± 0.05	0.94 ± 0.05	<0.01
Glucose (mmol/L)	5.10 ± 0.50	5.38 ± 0.84	0.02	4.99 ± 0.53	5.38 ± 0.83	<0.01
Insulin (uU/ml)	7.59 (5.33~11.59)	10.18 (6.69~14.49)	0.01	7.36 (5.45~10.68)	10.23 (6.69~15.53)	<0.01
HOMA-IR	1.76 (1.16~2.62)	2.41 (1.52~3.59)	<0.01	1.63 (1.11~2.35)	2.36 (1.51~3.57)	<0.01
SBP, mmHg	118.3 ± 14.32	121.1 ± 14.95	0.22	118.5 ± 15.00	119.8 ± 14.33	0.44
DBP, mmHg	75.29 ± 9.66	75.23 ± 10.36	0.96	74.91 ± 9.68	75.65 ± 9.39	0.43
TC (mmol/L)	4.68 ± 0.82	4.85 ± 0.94	0.24	4.61 ± 0.96	4.79 ± 0.97	0.11
TG (mmol/L)	1.42 ± 0.57	1.88 ± 1.11	<0.01	1.42 ± 0.89	1.83 ± 1.11	<0.01
HDL-C (mmol/L)	1.18 ± 0.35	1.08 ± 0.23	0.01	1.17 ± 0.29	1.10 ± 0.23	0.03
LDL-C (mmol/L)	3.04 ± 0.68	3.18 ± 0.81	0.25	3.02 ± 0.82	3.17 ± 0.84	0.15
ESS	7 (2~11)	9 (6~13)	<0.01	5.5 (2~10)	8 (4~11)	<0.01
AHI	2.41 (0.80~3.97)	23.10 (13.20~48.70)	<0.01	2.0 (0.97~3.43)	20.55 (11.58~38.89)	<0.01
Mean SaO_2_	96.26 ± 1.55	94.04 ± 2.98	<0.01	96.29 ± 1.29	94.42 ± 2.86	<0.01
Minimum SaO_2_	91.00 ± 9.86	78.19 ± 11.46	<0.01	92.23 ± 3.67	80.20 ± 10.57	<0.01
ODI	2.20 (0.80~4.70)	25.40 (13.60~50.30)	<0.01	2.10 (0.9~4.0)	23.30 (12.60~41.55)	<0.01
MAI	20.90 (12.60~30.40)	27.10 (14.60~42.00)	<0.01	17.7 (9.68~25.13)	26.05 (16.83~39.10)	<0.01

Normally distributed data were presented as means ± standard deviation (SD), medians (interquartile range) and categorical data were presented as the number (percentage).

Abbreviations: BMI, Body mass index; NC, neck circumference; WC, waist circumference, HC, hip circumference; HOMA-IR, homeostasis model of assessment for insulin resistence index; WHR, waist circumference/hip circumference ratio; SBP, systolic blood pressure; DBP, diastolic blood pressure; AHI, apnea-hypopnea index; SaO2, oxygen saturation; ODI, oxygen desaturation index; MAI, Micro-arousal index; ESS, Epworth sleepiness score; OSAS, obstructive sleep apnea syndrome; TC, total cholesterol; TG, triglyceride; HDL-C, high density lipoprotein cholesterol; LDL-C, low density lipoprotein cholesterol.

**Table 2:** Demographic and clinical characteristics of the enrolled subjects stratified by OSAS severity in both non-smokers and ever-smokers.

Variables	Ever-smokers					Non-smokers				
	Non-OSAS (N = 51)	Mild OSAS (N = 83)	Moderate OSAS (N = 96)	Severe OSAS (N = 108)	p value	Non-OSAS (N = 87)	Mild OSAS (N = 103)	Moderate OSAS (N = 100)	Severe OSAS (N = 91)	p value^#^
Age, years	38.65 ± 11.14	39.57 ± 9.28	42.65 ± 10.56	43.71 ± 9.72	<0.01	35.60 ± 10.98	40.40 ± 11.57	42.38 ± 12.97	41.19 ± 12.10	<0.01
BMI, Kg/m^2^	24.61 ± 3.01	25.78 ± 3.21	26.83 ± 2.88	27.78 ± 3.17	<0.01	23.83 ± 2.41	25.64 ± 3.02	26.09 ± 3.27	26.58 ± 2.89	<0.01
NC, cm	38.29 ± 2.40	39.16 ± 2.33	39.97 ± 2.45	40.59 ± 2.44	<0.01	37.82 ± 2.22	38.83 ± 2.68	39.33 ± 2.59	39.71 ± 2.31	<0.01
WC,cm	89.21 ± 7.89	92.63 ± 8.43	96.15 ± 7.59	98.89 ± 8.08	<0.01	86.86 ± 7.65	92.01 ± 7.61	94.11 ± 8.05	95.35 ± 7.74	<0.01
HC,cm	97.29 ± 5.56	98.74 ± 6.04	100.8 ± 5.62	102.2 ± 6.51	<0.01	96.45 ± 5.75	98.84 ± 6.39	100.0 ± 6.54	100.3 ± 5.81	<0.01
WHR	0.92 ± 0.04	0.94 ± 0.04	0.95 ± 0.05	0.97 ± 0.04	<0.01	0.90 ± 0.05	0.93 ± 0.05	0.94 ± 0.04	0.95 ± 0.04	<0.01
Glucose (mmol/L)	5.10 ± 0.50	5.25 ± 0.97	5.39 ± 0.79	5.47 ± 0.76	<0.01	4.99 ± 0.53	5.39 ± 0.88	5.38 ± 0.85	5.34 ± 0.74	<0.01
Insulin (uU/ml)	7.59 (5.33~11.59)	8.20 (5.83~12.24)	9.61 (6.94~13.52)	12.42 (8.02~19.29)	<0.01	7.36 (5.45~10.68)	9.37 (6.42~15.18)	10.51 (7.07~15.60)	10.33 (6.67~15.57)	<0.01
HOMA-IR	1.76 (1.16~2.62)	1.91 (1.26~2.75)	2.33 (1.60~3.30)	2.95 (1.90~4.83)	0.01	1.63 (1.11~2.35)	2.26 (1.43~3.50)	2.48 (1.61~3.47)	2.35 (1.49~3.91)	<0.01
SBP, mmHg	118.3 ± 14.32	122.7 ± 14.45	125.2 ± 17.07	126.3 ± 11.78	0.64	118.5 ± 15.00	122.4 ± 13.86	122.9 ± 15.78	123.5 ± 10.89	0.03
DBP, mmHg	75.29 ± 9.66	78.53 ± 10.03	80.21 ± 10.64	78.26 ± 5.43	<0.01	74.91 ± 9.68	78.67 ± 9.80	78.07 ± 9.40	79.56 ± 5.17	<0.01
TC (mmol/L)	4.68 ± 0.82	4.76 ± 0.96	4.90 ± 0.92	4.87 ± 0.95	0.63	4.61 ± 0.96	4.86 ± 1.04	4.79 ± 0.98	4.69 ± 0.88	0.16
TG (mmol/L)	1.43 ± 0.57	1.81 ± 1.18	1.89 ± 1.07	1.91 ± 1.09	<0.01	1.42 ± 0.89	1.78 ± 1.10	1.82 ± 1.13	1.88 ± 1.08	<0.01
HDL-C (mmol/L)	1.18 ± 0.35	1.14 ± 0.25	1.08 ± 0.24	1.04 ± 0.20	<0.01	1.17 ± 0.29	1.14 ± 0.26	1.09 ± 0.22	1.06 ± 0.21	<0.01
LDL-C (mmol/L)	3.04 ± 0.68	3.06 ± 0.78	3.25 ± 0.85	3.21 ± 0.79	0.86	3.02 ± 0.82	3.22 ± 0.92	3.23 ± 0.80	3.04 ± 0.77	0.09
ESS	7(2~11)	7.5(4~11)	8(5~12)	12(8~15)	<0.01	5.5(2~10)	7.5(4~10)	6(3~11)	9(4~13)	<0.01
AHI	2.43 ± 1.58	9.01 ± 2.84	21.94 ± 4.47	55.18 ± 14.58	<0.01	2.0(0.97~3.43)	9.92 ± 2.79	21.62 ± 4.52	54.30 ± 14.16	<0.01
Mean SaO_2_	96.20 ± 1.59	95.33 ± 1.65	94.99 ± 1.83	92.21 ± 3.64	<0.01	96.29 ± 1.29	95.75 ± 1.41	94.92 ± 1.71	92.36 ± 3.83	<0.01
Minimum SaO_2_	90.96 ± 9.77	86.30 ± 6.75	80.67 ± 9.04	69.74 ± 10.71	<0.01	92.23 ± 3.67	86.8 ± 5.62	81.27 ± 7.99	71.55 ± 11.43	<0.01
ODI	2.20 (0.80~4.70)	10.3 (6.9~14.4)	23.9 (17.33~33.63)	56.9 (45.1~70.2)	<0.01	2.10 (0.9~4.0)	10.7 (7.7~14.0)	24.4 (18.43~30.88)	56.3 (41.1~68.1)	<0.01
MAI	20.9 (12.6~30.4)	21.2 (14.29~35.5)	26.5 (15.9~33.9)	34.8 (14.5~52.6)	<0.01	17.7 (9.68~25.13)	20.8 (11.8~30.6)	28.0 (20.1~36.3)	36.6 (20.8~48.5)	<0.01

Normally distributed data were presented as means ± standard deviation (SD), medians (interquartile range) and categorical data were presented as the number (percentage).

^#^Tested using the polynomial linear trend test for continuous variables.

Abbreviations: BMI, Body mass index; NC, neck circumference; WC, waist circumference, HC, hip circumference; HOMA-IR, homeostasis model of assessment for insulin resistence index; WHR, waist circumference/hip circumference ratio; SBP, systolic blood pressure; DBP, diastolic blood pressure; AHI, apnea-hypopnea index; SaO2, oxygen saturation; ODI, oxygen desaturation index; MAI, Micro-arousal index; ESS, Epworth sleepiness score; OSAS, obstructive sleep apnea syndrome; TC, total cholesterol; TG, triglyceride; HDL-C, high density lipoprotein cholesterol; LDL-C, low density lipoprotein cholesterol.

### Dyslipidemia, insulin resistance, and OSAS severity

Dyslipidemia was significant among all subjects with mild OSAS (odds ratio [OR], 2.83; 95% confidence interval [CI], 1.59–5.03); however, this significance disappeared when mild OSAS was stratified by smoking status (Table 3). The risk of insulin resistance increased significantly with OSAS severity in the non- and ever-smoker groups (Table 4). However, this significance could be partly attributed to confounders, such as obesity (i.e., BMI and WHR).

**Table 3:** Odds ratios of dyslipidemia at different OSAS severity groups as compared to normal subjects.

Smoking status	AHI	Dyslipidemia		OR (95%CI)	p value^*^
		Yes	No		
Non-smokers	<5	56	31	Reference	
	5–14.9	75	29	1.43(0.78~2.64)	0.28
	15–29.9	68	31	1.21(0.66~2.24)	0.54
	≥30	68	23	1.64(0.86~3.12)	0.15
Ever-smokers	<5	38	13	Reference	
	5–14.9	58	25	0.79(0.36~1.74)	0.69
	15–29.9	75	21	1.22(0.55~2.70)	0.68
	≥30	86	22	1.34(0.61~2.93)	0.54
All subjects	<5	94	44	Reference	
	5–14.9	133	54	2.83(1.59~5.03)	<0.01
	15–29.9	144	52	1.30(0.80~2.09)	0.33
	≥30	154	45	1.39(0.86~2.23)	0.18

Abbreviations: OSAS, obstructive sleep apnea syndrome; AHI, apnea-hypopnea index; CI, confidence interval.

^*^p value was calculated from Pearson χ^2^ test.

**Table 4:** Odds ratios of insulin resistance at different OSAS severity groups as compared to normal subjects. *p value was calculated from Pearson χ^2^ test.

Smoking status	AHI	Insulin resistance		OR (95%CI)	p value
		Yes	No		
Non-smokers	<5	15	72	Reference	
	5–14.9	39	65	2.88(1.45~5.70)	0.002
	15–29.9	43	56	3.69(1.86~7.30)	<0.001
	≥30	36	47	4.39(2.20~8.78)	<0.001
Ever-smokers	<5	11	40	Reference	
	5–14.9	20	63	1.15(0.50~2.66)	0.736
	15–29.9	36	60	2.18(1.00~4.78)	0.049
	≥30	57	51	4.06(1.89~8.75)	<0.001
All subjects	<5	26	112	Reference	
	5–14.9	59	128	1.99(1.17~3.36)	0.010
	15–29.9	79	116	2.19(1.29~3.72)	0.003
	≥30	93	106	3.78(2.27~6.29)	<0.001

Abbreviations: OSAS, obstructive sleep apnea syndrome; AHI, apnea-hypopnea index; CI, confidence interval.

### Metabolic parameters in the OSAS and smoking groups

Significant correlations were found between almost all metabolic profiles and sleep parameters (data not shown). Even after adjusting for age, BMI, WHR, and ESS, significant correlations were detected between ODI and HDL-C (r = −0.080; *p* = 0.048), MAI and LDL-C (r = 0.101; *p* = 0.008); insulin level and AHI (r = 0.094, *p* = 0.012), ODI (r = 0.122, *p* = 0.001) and MAI (r = 0.078, *p* = 0.038); and HOMA-IR and AHI (r = 0.084, *p* = 0.025) and ODI (r = 0.109, *p* = 0.004). However, when we stratified by smoking status and adjusted for age, BMI, WHR and ESS, only insulin level (r = 0.198, *p* = 0.001; r = 0.196, *p* = 0.001, respectively) and HOMA-IR (r = 0.193, *p* = 0.001; r = 0.186, *p* = 0.002, respectively) correlated positively with AHI and ODI in smoking patients with OSAS. In addition, we performed multiple linear regression analyses to evaluate independent relationships between metabolic variables and selected factors in smoking patients with OSAS. After adjusting for multiple confounding factors, we found that age affected glucose and TG, while obesity mostly affected glucose metabolism (Supplementary Table 1).

### Effect of the interaction between OSAS and smoking on metabolic parameters including the lipid profile

Most of the metabolic parameters were aberrant in OSAS smokers compared with the other three groups, indicating that OSAS smokers might suffer from more severe metabolic disorders. Strong main effects of OSAS were detected on glucose (F = 6.515, *p* < 0.001), insulin (F = 9.518, *p* < 0.001), HOMA-IR (F = 8.771, *p* < 0.001), TG (F = 6.183, *p* < 0.001), and HDL levels (F = 8.108, *p* < 0.001). However, these effects disappeared after adjusting for confounding factors, such as age, BMI, WHR, and ESS. An interaction between OSAS and smoking for metabolic variables was found only for the insulin level (F = 4.144, *p* = 0.006) and HOMA-IR (F = 3.123, *p* = 0.025) (Table 5).

**Table 5:** Markers of metabolic outcomes in never-smoking and ever-smoking OSAS patients.

Variable	Never-smoking		Ever-smoking		p value^&^	Main Effects (p value)^*^		Interaction Effects (p value)^*^	Main Effects (p value)^#^		Interaction Effects (p value)^#^
	AHI < 5	AHI ≥ 5	AHI < 5	AHI ≥ 5		AHI	Smoking	AHI × Smoking	AHI	Smoking	AHI × Smoking
Glucose (mmol/L)	4.99 ± 0.53	5.38 ± 0.83	5.10 ± 0.50	5.38 ± 0.84	<0.001	<0.001	0.740	0.350	0.181	0.754	0.745
Insulin (uU/ml)	8.48 ± 4.52	11.79 ± 7.56	9.34 ± 6.58	12.28 ± 8.23	0.001	<0.001	0.477	0.003	0.382	0.576	0.006
HOMA-IR	1.90 ± 1.08	2.92 ± 2.47	2.13 ± 1.51	3.01 ± 2.29	0.001	<0.001	0.624	0.007	0.450	0.493	0.025
TC (mmol/L)	4.61 ± 0.96	4.79 ± 0.97	4.68 ± 0.82	4.85 ± 0.94	0.330	0.274	0.367	0.527	0.580	0.412	0.623
TG (mmol/L)	1.42 ± 0.89	1.83 ± 1.11	1.43 ± 0.57	1.88 ± 1.11	<0.001	<0.001	0.668	0.991	0.255	0.824	0.795
HDL-C (mmol/L)	1.17 ± 0.29	1.10 ± 0.23	1.18 ± 0.35	1.08 ± 0.23	0.012	<0.001	0.762	0.949	0.104	0.558	0.975
LDL-C (mmol/L)	3.02 ± 0.82	3.17 ± 0.84	3.04 ± 0.68	3.17 ± 0.81	0.231	0.131	0.853	0.270	0.250	0.951	0.341

Abbreviations: HOMA-IR, homeostasis model of assessment for insulin resistance index; AHI, apnea-hypopnea index; OSAS, obstructive sleep apnea syndrome; TC, total cholesterol; TG, triglyceride; HDL-C, high density lipoprotein cholesterol; LDL-C, low density lipoprotein cholesterol.

^&^p value indicates overall comparison.

^*^p value without adjustment.

^#^p value adjusted for age, BMI,WHR and ESS.

### Effects of cessation of smoking on metabolic parameters

The effects of stopping smoking on metabolic parameters were also assessed. No differences were found in glucose, insulin, HOMA-IR, TC, TG, or HDL-C in the current smoker (n = 279) and ex-smoker groups (n = 59), compared with never-smokers (n = 381). Current smokers had higher glucose, insulin, HOMA-IR, TC, and TG, whereas the HDL-C level was decreased compared with the other groups, but the differences were not significant, according to the *p*-values shown in Table 6. Serum glucose, insulin, and lipid levels were the same between patients with OSAS who quit smoking (n = 59) and subjects who had never smoked. Of all metabolic parameters, LDL-C seemed to benefit most in patients with OSAS who quit smoking (Table 5). However, this significance could be due to confounding factors, such as obesity (i.e., BMI and WHR).

**Table 6:** Metabolic variables of OSAS subjects with different smoking status.

	Never-smokers (n = 381)	Current smokers (n = 279)	Ex-smokers (n = 59)	p value
Glucose (mmol/L)	5.29 ± 0.79	5.34 ± 0.76	5.30 ± 0.99	0.714
Insulin (uU/ml)	11.05 ± 7.11	11.97 ± 8.08	11.20 ± 7.99	0.302
HOMA-IR	2.69 ± 2.27	2.91 ± 2.20	2.73 ± 2.27	0.453
TC (mmol/L)	4.74 ± 0.97	4.85 ± 0.95	4.68 ± 0.82	0.241
TG (mmol/L)	1.73 ± 1.07	1.83 ± 1.06	1.71 ± 1.03	0.463
HDL-C (mmol/L)	1.12 ± 0.25	1.09 ± 0.25	1.16 ± 0.28	0.093
LDL-C (mmol/L)	3.13 ± 0.83	3.21 ± 0.81	2.91 ± 0.65	0.031

Abbreviations: HOMA-IR, homeostasis model of assessment for insulin resistence index; OSAS, obstructive sleep apnea syndrome; TC, total cholesterol; TG, triglyceride; HDL-C, high density lipoprotein cholesterol; LDL-C, low density lipoprotein cholesterol.

p value indicates overall comparison.

## Discussion

This was the first study to assess the relative impact of OSAS and smoking on metabolic disorders. We found that both OSAS and smoking were major risk factors for metabolic disorders, and conferred an additional risk on glucose abnormality. Patients with OSAS might experience minor benefits from quitting smoking.

Both dyslipidemia and insulin resistance are precursors to atherosclerosis and CVD. One study reported that OSAS combined with dyslipidemia conferred additional adverse effects with respect to the prevalence of atherosclerotic CVD^27^. Thus, exploring factors that affect metabolic variables will help in the development of therapeutic strategies to lower cardiovascular risks. The detrimental effects of OSAS in patients with dyslipidemia have been well-documented. More patients with OSAS have dyslipidemia than subjects without OSAS, and only LDL-C is independently associated with OSAS^6^. CPAP treatment significantly decreases the TC level, with slight changes in TG, HDL, and LDL-C levels^7^. CPAP-treated OSAS patients were shown to have lower cardiovascular mortality (hazard ratio, 0.37; 95% CI, 0.16–0.54) than did untreated OSAS subjects^28^. TG/HDL-C abnormalities have been suggested to be related to insulin resistance. In our study, we found that patients with OSAS had a higher risk of glucose abnormalities. Therefore, we propose that insulin resistance is potentially a key mediator of the relationship between smoking and CVDs.

Insulin resistance is often considered to be aggravated by smoking. The mechanisms of smoking-induced insulin resistance have not been clearly shown. Cigarette smoke contains more than 4,000 chemicals, heavy metals, and free radicals, and approximately 400 are considered harmful^29^. Smoke dispersed in the circulation through blood-gas exchange at the lung triggers a cascade of oxidative stress and systemic inflammation biomarkers, after which hormonal, biochemical, and metabolic disorders may appear. In addition, nicotine, the most toxic substance in smoking, impairs hepatic lipase function, which can lead to atherogenic insulin resistance^30^.

Smoking and OSAS have a bidirectional relationship. Smoking induces narrowing of the oropharynx in the upper airway by increasing edema and thickness of the uvular mucosa, which may be the most common mechanism underlying the increase in OSAS severity^31^. Furthermore, several other mechanisms might also explain how smoking causes and aggravates OSAS, by (1) changing the sleep architecture; (2) relaxing the upper airway muscles and reducing upper airway neural reflex sensitivity; and (3) increasing the sleep arousal threshold^4^. In addition, many patients with OSAS smoke. Chronic hypoxia, which is a characteristic of OSAS, may be responsible for the higher number of nicotine binding sites observed in smokers due to adaptation^32^. The increase in the number of nicotine binding sites further perpetuates the vicious cycle of smoking and increased smoking frequency^4^. One study reported a synergistic effect between cigarette smoking and OSAS, which increased cardiovascular risk^19^. Our study also confirmed that the combined effect of smoking and OSAS disturbed glucose metabolism. Thus, patients with OSAS should be aware of this association.

An increase in HDL-C after cessation of smoking was reported in patients with diabetes in a recent meta-analysis^33^. An improved lipid profile after cessation of smoking has also been confirmed in the general population^15^. In our study, we found only trends to slight improvement, without statistical significance, in the serum lipid profile and glucose metabolism. This might be partly explained by the moderate weight gain that often occurs in patients who quit smoking. Taken together, although dyslipidemia and abnormal glycometabolism seem to improve only slightly in people who quit smoking, this benefit should nevertheless be emphasized.

Some limitations of this study should be addressed. First, inherent deficits in the cross-sectional design prevented the establishment of causality regarding the combined effects of OSAS and smoking on metabolic disorders. Second, some significance might be partly attributed to confounding factors, such as BMI and WHR; some other potential variables could exist, although we did adjust for potential confounding factors. Third, we only assessed smoking exposure using a self-report questionnaire. Unfortunately, this would give us only partial information on smoking duration and intensity in former smokers. Fourth, we only recruited men, so the conclusions might not be generalizable to the general population. Fifth, although we excluded patients with certain sleep disorders, we could not exclude sleep disorders such as insomnia based on one-night PSG monitoring. Finally, all participants were recruited from two of the largest sleep centers (in Soochow and Shanghai) in China, and not from the community, which might also constitute potential selection bias.

In conclusion, this is the first study to explore the combined effects of OSAS and smoking on metabolic variables. Interactions between smoking and OSAS aggravated metabolic disorders after multiple adjustments. Our findings will help doctors encourage their patients to quit smoking, not only to improve OSAS, but also to prevent metabolic disorders and further cardiovascular morbidities.

### Ethical approval

The ethics committee of Shanghai Jiao Tong University Affiliated Sixth People’s Hospital and The Second Affiliated Hospital of Soochow University, approved the study. All the participants have given the informed consent before taking part in the study.

## Electronic supplementary material

Smoking Questionnaire and Supplementary table 1
