# Supplementary material for: Smoking, obstructive sleep apnea syndrome and their combined effects on metabolic parameters: Evidence from a large cross-sectional study
Source: Sci Rep. 2017 Aug 18;7:8851. doi: 10.1038/s41598-017-08930-x (PMC5562758; doi:10.1038/s41598-017-08930-x)
Supplement: Supplementary file 1 — Smoking Questionnaire and Supplementary table 1 [file 41598_2017_8930_MOESM1_ESM.pdf]

Smoking, obstructive sleep apnea syndrome and their combined effects on metabolic parameters: Evidence from a large cross-sectional study

Huaming Zhu MD[1,2,3,4*], Huajun Xu MD,Ph D[3,4*], Rui Chen [#] MD,Ph D [1,2], Suru Liu MD,Ph D[3,4], Yunyan Xia MD [3,4], Yiqun Fu MD[3,4], Xinyi Li MD[3,4], Yingjun Qian MD,Ph D [3,4], Jianyin Zou MD,Ph D, Hongliang Yi [#] MD, Ph D[3,4], Jian Guan MD,Ph D [3,4]

1 Department of Respiratory Medicine, the Second Affiliated Hospital of Soochow University, 1055 Sanxiang Road, 215004, Suzhou, China.

2 Sleep Center, The Second Affiliated Hospital of Soochow University, 1055 Sanxiang Road, 215004, Suzhou, China

3 Department of Otolaryngology Head and Neck Surgery & Center of Sleep Medicine, Shanghai Jiao Tong University Affiliated Sixth People's Hospital, 600 Yishan Road, 200233 Shanghai, China.

4 Otolaryngological Institute of Shanghai Jiao Tong University, 600 Yishan Road, 200233 Shanghai, China.

*Both Huaming Zhu and Huajun Xu contributed equally to this work

Corresponding author: Rui Chen MD,Ph D (E-mail: chenruigood@126.com) and Hongliang Yi MD,Ph D (yihongl@126.com).

Smoking Questionnaire

Name: gender: Date of birth (day/month/year):

1) smoking status--(A never B current C past)

If you choose B, please answer the following questions:

2) age when smoking started---( )

3) the duration of smoking (years) --( )

4) reasons for starting smoking---( )

5) average number of cigarettes smoked per day---------------------------------( )

6) cessation attempts and their forms---( )

7) reasons for trying to quit--( )

If you choose C, please answer the following questions:

8) age when smoking started---( )

9) the duration of past smoking (years) --( )

10) quitting years of exsmokers---( )

11) average number of cigarettes smoked per day in the past-------------------( )

12) cessation attempts and their forms---( )

13) quit success--( )

Supplementary table 1 Multivariate linear regression model of selected factors and metabolic variables in smoking patients with OSAS

**Variables**	**Glucose**		**Insulin**		**HOMA-IR**		**TC**		**TG**		**HDL-C**		**LDL-C**	
	β	p	β	p	β	p	β	p	β	p	β	p	β	p
**Age, years**	0.230	<0.01	-0.075	0.145	-0.029	0.579	0.042	0.498	-0.134	0.024	0.107	0.070	0.098	0.107
**BMI, Kg/m2**	0.190	0.009	0.385	<0.01	0.395	<0.01	0.119	0.113	0.214	0.003	-0.097	0.178	0.044	0.554
**WHR**	-0.008	0.911	0.060	0.316	0.046	0.447	-0.107	0.130	0.027	0.692	-0.147	0.031	-0.051	0.464
**ESS**	0.014	0.822	0.036	0.500	0.023	0.671	-0.052	0.416	-0.025	0.681	-0.050	0.412	-0.022	0.729
**AHI**	-0.072	0.571	0.057	0.608	0.048	0.668	0.052	0.694	-0.072	0.570	-0.051	0.683	0.062	0.636
**LSpO2**	0.082	0.302	0.036	0.605	0.033	0.640	-0.036	0.658	-0.135	0.091	0.054	0.496	0.121	0.140
**ODI**	0.149	0.240	0.182	0.099	0.171	0.129	-0.013	0.919	-0.051	0.689	-0.028	0.824	0.085	0.515
**MAI**	0.001	0.998	-0.017	0.756	-0.023	0.677	0.043	0.516	0.051	0.418	0.072	0.256	0.088	0.177

Abbreviations: BMI, Body mass index; HOMA-IR, homeostasis model of assessment for insulin resistence index; WHR, waist circumference/hip circumference ratio; AHI, apnea-hypopnea index; SaO2, oxygen saturation; ODI, oxygen desaturation index; MAI, Micro-arousal index; ESS, Epworth sleepiness score; OSAS, obstructive sleep apnea syndrome; TC, total cholesterol; TG, triglyceride; HDL-C, high density lipoprotein cholesterol; LDL-C, low density lipoprotein cholesterol
